# Purified Serum IgG from a Patient with Anti-IgLON5 Antibody Cause Long-Term Movement Disorders with Impaired Dopaminergic Pathways in Mice

**DOI:** 10.3390/biomedicines11092483

**Published:** 2023-09-07

**Authors:** Yining Gao, Hongxia Li, Huoqing Luo, You Ni, Yifan Feng, Lu He, Qinming Zhou, Ji Hu, Sheng Chen

**Affiliations:** 1 Department of Neurology, Ruijin Hospital, Shanghai Jiao Tong University School of Medicine, Shanghai 200023, China; gynsjtu@163.com (Y.G.); hongxialee2017@163.com (H.L.); 22111220040@m.fudan.edu.cn (Y.N.); s_hlxj@163.com (L.H.); zqmm2005@163.com (Q.Z.); 2 School of Life Science and Technology, ShanghaiTech University, Shanghai 201210, China; luohq@shanghaitech.edu.cn (H.L.); fengyf@shanghaitech.edu.cn (Y.F.); 3 Co-Innovation Center of Neuroregeneration, Nantong University, Nantong 226007, China

**Keywords:** anti-IgLON5 disease, movement disorders, neuropathology, dopaminergic pathways, substantia nigra pars compacta

## Abstract

**Background**: Anti-IgLON5 disease is a rare autoimmune disease of the central nervous system. It typically manifests as a chronic condition, characterized by cognitive impairments, movement disorders, and sleep disorders. The mechanisms underlying movement disorders in this disease remain poorly understood due to a lack of research. Furthermore, this disease exhibits both neuroimmune and neurodegenerative characteristics. The objective of this study is to explore the underlying mechanisms of movement disorders caused by anti-IgLON5 antibodies for the first time. **Methods**: Antibodies were purified from the serum of a confirmed patient of anti-IgLON5 disease. The passive transfer animal models were employed, where antibodies were continuously injected into the substantia nigra pars compacta (SNc) of the mouse midbrain using stereotactic injection to explore the mechanism of movement disorder. The effects of anti-IgLON5 antibodies on dopaminergic neurons in the SNc and neurodegeneration were examined through immunohistochemistry. Changes in neurotransmitter levels in the basal ganglia were assessed using high-performance liquid chromatography. Additionally, RNA-seq was employed to identify the differentially expressed genes associated with the short-term and long-term effects of anti-IgLON5 antibody on the SNc. **Results**: Mice injected with anti-IgLON5 antibodies in the SNc exhibited persistent movement impairments for up to 3 months. One week after antibody injection, the number of TH neurons significantly decreased compared to the control group, accompanied by reduced projection fibers in the basal ganglia and decreased dopamine levels. After 3 months of antibody injection, an increase in phosphorylated Tau was observed in the SNc of the midbrain. Additionally, long-term sustained activation of microglia was detected in the SNc. The differentially expressed genes of long-term effects of IgLON5 antibodies were different from their short-term effects on the SNc. **Conclusion**: Purified serum IgG from a patient with anti-IgLON5 antibodies can cause long-term movement disorder in mice. The movement disorders appear to be linked to the impaired dopaminergic pathway, and the increased p-Tau showed neurodegenerative changes induced by the anti-IgLON5 antibody.

## 1. Introduction

Anti-IgLON5 disease is an autoimmune encephalitis mediated by anti-IgLON5 antibodies. It was first reported by Sabater et al. in 2014 [1]. Unlike other encephalitis disorders, anti-IgLON5 disease presents with a chronic and long-term onset [2]. Its primary clinical symptoms include cognitive impairments, movement disorders, and sleep disorders [3]. Our previous studies have explored the mechanisms of anti-IgLON5 antibody-mediated cognitive impairments [4], but there is still a lack of understanding regarding the underlying mechanisms of movement disorders in this disease. Moreover, autopsy results of patients with anti-IgLON5 disease showed high levels of phosphorylated Tau protein, primarily affecting the hypothalamus and brainstem [5], indicating that anti-IgLON5 disease exhibits both neurodegenerative and neuroimmune characteristics. This study aims to establish the first mouse model of anti-IgLON5 antibody-mediated movement disorders and conduct longitudinal observations. Additionally, we will investigate the relationship between phosphorylated Tau protein and anti-IgLON5 disease in vivo for the first time.

Recent imaging studies have observed decreased striatal uptake in SPECT of patients with anti-IgLON5 disease, indicating alterations in the dopaminergic pathway [6,7,8]. ^18^F-FDG PET has shown increased metabolism in the basal ganglia, cerebellum, and brainstem in some patients [9,10,11,12]. It is speculated that the movement symptom phenotype in anti-IgLON5 disease may be associated with the dopaminergic pathway between the SNc and striatum. The nigrostriatal pathway is one of the main pathways in the brain that controls movement [13,14]. In the nigrostriatal pathway, the projection from the SNc to the striatum is mainly mediated by dopamine neurons [15]. These neurons release dopamine as a neurotransmitter and regulate striatal activity through interactions with dopamine D1 and D2 receptors on GABAergic neurons in the striatum [16,17]. Abnormal activity of SNc neurons or insufficient dopamine levels can lead to movement disorders [18].

In this study, we utilized passive immunization by infusing antibodies into the SNc of mice to establish a stable mouse model of movement disorders associated with anti-IgLON5 disease. We employed stereotaxic injection techniques, motor behavior tests, immunohistochemistry, high-performance liquid chromatography (HPLC), whole transcriptome gene sequencing, Western blotting (WB), and other experimental techniques to explore the underlying mechanisms of behavioral changes in mice. Additionally, we aimed to provide new insights and clues for the treatment of anti-IgLON5 diseases in clinical settings.

## 2. Materials and Methods

### 2.1. Animal

Male C57BL/6 mice aged 7–8 weeks were obtained from Charles River (Beijing, China). The animals were handled according to institutional guidelines and governmental regulations for animal care and use. The mice were housed in groups of 5 per cage and maintained under a 12 h light–dark cycle (lights on from 9:00 p.m. to 9:00 a.m.) at a temperature of 22–25 °C. They had free access to food and water. All in vivo and in vitro experiments were conducted in compliance with the approved protocols of the Institutional Animal Care and Use Committee (IACUC) at both Shanghai Jiaotong University and ShanghaiTech University.

### 2.2. Human IgG Purification

We purified IgG from the serum samples of a 74-year-old female patient with anti-IgLON5 disease and a healthy individual of the same age and sex (healthy control IgG) (Supplementary Figure S1). Protein A was used for the purification process [19]. The concentration of the purified IgG from the patient’s sample was 2.3 mg/mL, while the concentration of the healthy control IgG was 1.8 mg/mL.

### 2.3. Stereotactic IgG Injection

Daily stereotactic injections of purified patient IgG or control IgG were performed over a period of 7 days into the SNc of C57BL/6 male mice [19,20,21]. The mice were adequately anesthetized with a mixture of 1.5% isoflurane and oxygen, and their heads were fixed onto a stereotactic apparatus. A small midline incision was made to expose the skull, and the injection sites within the SNc were marked and drilled. Using a micro-syringe pump (Nanoject III #3-000-207, DRUMMOND, Broomall, PA, USA), 1 μL of purified patient or control IgG was injected daily into the SNc at the coordinates AP −3.10 mm, ML ±1.25 mm, DV +4.50 mm. The injections were delivered in 50 steps, with 20 nL injected every 30 s. Following each injection, the injector remained stationary for an additional 10 min to allow the IgG to diffuse.

### 2.4. Behavioral Tests

Before the commencement of the behavioral experiments, the experimenters need to handle the mice for three consecutive days to acclimate them to the experimental procedures and minimize the impact of their stress responses on the behavioral outcomes. Each handling session lasts for 5–10 min.

### 2.5. Open Field Test (OFT)

The OFT is used to assess the locomotor activity and anxiety levels of mice. The open field area is an opaque hollow box measuring 38 × 38 × 40 cm. Prior to the start of the experiment, the test mice were placed into the open field from the same angle, and the data collection software was initiated. The typical duration of data collection was 5 min, during which an infrared camera and software were used to track the mice’s movement trajectories [22].

### 2.6. Pole Test (PT)

The PT is used to assess the motor coordination of mice. In the experiment, a round metal pole with a diameter of approximately 1 cm and a length of 50 cm is used. The top of the pole is fixed with a round ball with a diameter of approximately 1 cm, which serves as the starting position for the experiment. Prior to conducting the pole test, the mice were given two days to acclimate to the pole apparatus and learn the method of climbing the pole. The acclimation process is the same as the experimental procedure. The mice were gently placed on the top of the ball, the timer was started, and the time taken for the mice to descend and have all four paws touch the ground was recorded. The experiment was performed three times, with a minimum interval of 30 min between each trial. Finally, the average of the three trials was calculated to obtain the final result [23].

### 2.7. Beam Balance Test (BBT)

The BBT is used to assess the motor balance ability of mice. The beam is a narrow wooden stick measuring 80 cm in length and approximately 0.8 cm in width. It is elevated 50 cm above the ground and positioned parallel to the ground. The starting end of the beam was illuminated by a bright desk lamp, while the endpoint consisted of a dark chamber made of black acrylic panels. Inside the chamber, familiar bedding and food for the mice are provided to test their balance and motor abilities. Prior to conducting the beam walking test, a learning and adaptation phase lasting three days was carried out. During this phase, the mice were first familiarized with the black chamber at the endpoint. In the actual beam walking experiment, the timer was started as the mouse departed from the starting point, and the time taken for the mouse to reach the endpoint and the number of paw slips during this period were measured. The experiment was repeated three times, with a rest period of at least 30 min between each trial. The two parameters, time and paw slips, are averaged separately from the three trials to obtain the final results [24].

### 2.8. Histology and Imaging

To prepare the mice for analysis, they were deeply anesthetized by injecting tribromoethanol into the abdominal cavity. Saline was then perfused through the heart to remove most of the blood. The brain was carefully removed and fixed with 4% paraformaldehyde (PFA). After overnight fixation, the brain was transferred to a 30% sucrose solution and kept at 4 °C for 48 h. Coronal brain sections, with a thickness of 40 μm, were obtained using a cryostat microtome (Leica CM3050S, Leica Biosystems, Wetzlar, Germany). These sections were blocked using a buffer containing 5% bovine serum albumin (BSA) and 0.3% Triton X-100 in 1×PBS. Primary antibodies, such as anti-NeuN (1:1000, #26975-1-AP, Proteintech, Rosemont, IL, USA), anti-tyrosine hydroxylase (1:1000, #AB9702, Sigma, St. Louis, MO, USA) and anti-IBA1 (1:1000, #019-19741, Wako, Richmond, VA, USA), were applied to the sections and incubated for 48 h at 4 °C. Subsequently, secondary antibodies, including AlexaFluor 488 goat anti-chicken IgG (1:1000; #A-21467, Thermo Fisher, Waltham, MA, USA) and AlexaFluor 594 donkey anti-rabbit IgG (1:1000, #R-37119, Thermo Fisher, Waltham, MA, USA), were applied and incubated for 2 h at room temperature.

After each antibody incubation, the sections were washed with 1×PBS buffer to remove any excess antibodies. DAPI staining was used to identify cell bodies, and the slides were sealed with 10% glycerine. Fluorescent images were captured using microscopy, and the analysis was performed using ImageJ and Qupath software.

### 2.9. Western Blot Analysis

Mouse SNc tissues were subjected to Western blotting using established protocols. Equal amounts of protein were separated by SDS-polyacrylamide gel electrophoresis (10%) and transferred to nitrocellulose membranes. After blocking with 5% skim milk powder in TBST (Tris-buffered saline with 0.05% Tween 20) for 2 h at 25 °C, the membranes were incubated overnight with primary antibodies against Tau (1:3000, #GB11178, Servicebio, Wuhan, China), p-Tau (1:2000, MN1020, Invitrogen, Carlsbad, CA, USA), and actin (1:2000, GB15001, Servicebio, Wuhan, China). Subsequently, the membranes were washed thrice with TBST for 15 min and incubated with secondary antibodies (ZSGB-BIO, Beijing, China) in 5% skim milk powder/TBST. Following that, the membranes were exposed to BCIP/NBT alkaline phosphatase color-developing reagent (Beyotime Institute of Biotechnology, Shanghai, China) for 15 min. The protein bands of interest were scanned, and band density was analyzed using the Quantity One automatic imaging analysis system (Bio-Rad, Hercules, CA, USA) [25].

### 2.10. RNA Extraction and RNA Sequencing

After euthanasia, SNc tissue was dissected and immediately collected into RNase-free tubes, followed by freezing at −80 °C until analysis. Total RNA extraction and purification were performed using TRIzol reagent (Invitrogen, Carlsbad, CA, USA). The quantity and purity of RNA from each sample were assessed using NanoDrop ND-1000 (NanoDrop, Wilmington, DE, USA). RNA integrity was evaluated using the Bioanalyzer 2100 (Agilent, Santa Clara, CA, USA), ensuring a RIN number >7.0, and confirmed by denaturing agarose gel electrophoresis. LC-Bio, Hangzhou, China, conducted transcriptome-seq. Approximately 10 µg of total RNA was purified, and poly(A) RNA was isolated. The purified poly(A) RNA was fragmented, followed by cDNA library construction. The library underwent screening, and purification using UDG enzyme digestion, and PCR amplification to obtain the final sequencing library. The qualified library was sequenced on Illumina Hiseq4000 following recommended protocols. Differentially expressed mRNAs were identified using R packages edgeR or DESeq2, with fold changes ≥2 or ≤0.5 and a *p*-value < 0.05. Gene Ontology (GO) and Kyoto Encyclopedia of Genes and Genomes (KEGG) were used for enrichment analysis of differentially expressed mRNAs [26].

### 2.11. High-Performance Liquid Chromatography

Using a Welch Ultimate XB-C18 chromatographic column with dimensions of 150 × 4.6 mm and a particle size of 5 µm, the flow rate was set at 0.8 mL/min. The mobile phase consisted of 0.1% formic acid/water (aqueous phase) and 0.1% formic acid/acetonitrile (organic phase). Methanol was used as the needle wash solution, and the column temperature was set at 40 °C in the column oven. Standard substances were accurately weighed and prepared as a stock solution with a concentration of 2.00 mg/mL for later use. Then, pure methanol was sequentially diluted to prepare a series of standard curve working solutions with different concentrations. Samples were weighed and recorded, followed by the addition of a certain amount of methanol solution containing 0.1% formic acid: 300 µL for the SNc group and 500 µL for the Cpu group. Next, zirconium oxide grinding beads were added to the samples for 5 min of grinding, followed by 5 min of shaking using a vortex shaker. Subsequently, the samples were centrifuged at 13,000 rpm for 10 min, and the supernatant was collected and filtered through a 0.22 µm membrane filter. The filtrate was diluted 20 times for dopamine detection.

### 2.12. Statistical Analyses

All statistical analyses and graph plotting were performed using GraphPad Prism 8. When comparing two groups of data, we utilized the unpaired *t*-test. If the calculated *p*-value was less than 0.05, we considered it to indicate a statistically significant difference between the two groups. To visually represent the statistical significance, we used asterisks (*) to denote the level of significance. 

## 3. Results

### 3.1. The Patients’ Anti-IgLON5 Antibodies Induce Motor Disorders in Mice

**Figure 1:**
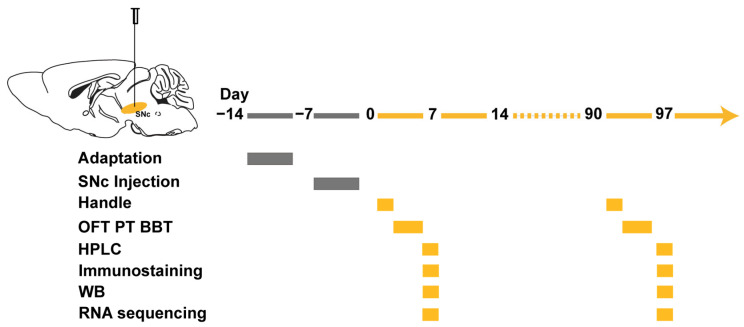
Flowchart for exploring the mechanism of anti-IgLON5 antibody-mediated movement disorders. SNc, substantia nigra compacta; OFT, open field test; PT, pole test; BBT, beam balance test; HPLC, high-performance liquid chromatography; WB, Western blot. After a 7-day adaptation period, the anti-IgLON5 group and the control group of mice were injected bilaterally into the SNc with anti-IgLON5 antibody or control IgG. Subsequently, the mice were subjected to behavioral tests for locomotor activity, HPLC, WB, and RNA-seq analysis at 7 days and 3 months (97th day) after the injection. Gray indicates the state before injection, while orange indicates the state after antibody injection.

**Figure 2:**
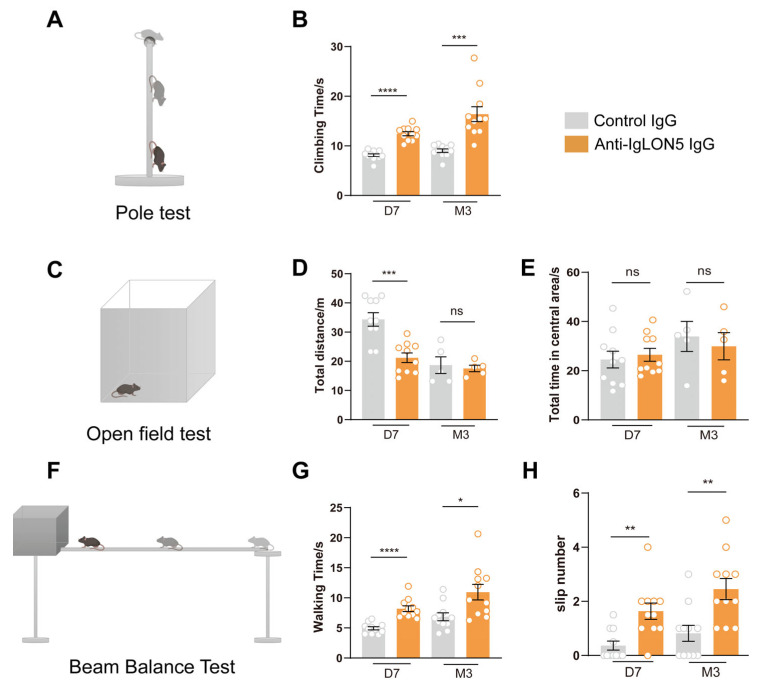
Movement disorders mediated by anti-IgLON5 antibodies. (**A**) Diagram of pole test (**B**) Pole test: Mice injected with anti-IgLON5 IgG exhibited decreased motor balance ability at 7 days (D7) (*n* = 10, *p* < 0.0001, *t*-test); this decline in motor balance ability persisted at 3 months (M3) (*n* = 10, *p* < 0.001, *t*-test). (**C**) Diagram of open field test. (**D**,**E**) Open field test: Mice injected with anti-IgLON5 IgG showed a significant decrease in motor ability at 7 days (*n* = 10, *p* < 0.001, *t*-test); no statistical difference was observed between the two groups after 3 months. (**F**) Diagram of beam balance test. (**G**,**H**) Beam balance test: Mice injected with anti-IgLON5 IgG showed increased time required for beam traversal at 7 days (*n* = 10, *p* < 0.0001, *t*-test), as well as an increase in the number of slips (*n* = 10, *p* < 0.001, *t*-test); these phenotypes persisted at 3 months, with prolonged beam traversal time (*n* = 10, *p* < 0.05, *t*-test) and an increased number of slips (*n* = 10, *p* < 0.01, *t*-test).* represents a *p*-value less than 0.05, ** represents a *p*-value less than 0.01, *** represents a *p*-value less than 0.001, and **** represents a *p*-value less than 0.0001. “ns” stands for no statistically significance.

To investigate the effects of anti-IgLON5 antibodies on mouse motor behavior, we employed a passive transfer approach by injecting patient IgG or control IgG into the bilateral SNc region to establish an animal model (Figure 1). In this study, the experimental and control group antibodies were purified from serum samples of a confirmed patient of anti-IgLON5 disease from Ruijin Hospital and a healthy individual with no detectable neuronal autoantibodies, respectively. Behavioral tests were conducted at 7 days post-injection (days 4–7) and at the 3-month time point to explore the long-term effects of the antibodies. During days 4–7, the open field test, beam balance test, and pole test revealed significant differences between the experimental and control groups. Mice injected with anti-IgLON5-IgG exhibited decreased locomotor activity in the OFT, increased number of slips and prolonged walking time in the BBT, and increased time to climb in the PT (OFT: *n* = 10, *p* < 0.001; BBT: *n* = 10, *p* < 0.0001; PT: *n* = 10, *p* < 0.0001). Mice injected with anti-IgLON5-IgG exhibited a noticeable decrease in total distance traveled and impaired balance, indicating the induction of short-term motor impairments by antibody injection. Importantly, the decline in motor balance persisted up to the 3-month time point in mice infused with anti-IgLON5-IgG, suggesting a relatively long-lasting effect of anti-IgLON5 antibodies (OFT: *n* = 10, *p* > 0.05; BBT: *n* = 10, *p* < 0.01; PT: *n* = 10, *p* < 0.001) (Figure 2).

### 3.2. The Anti-IgLON5 Antibodies in Patients Lead to a Reduction in SNc TH Neurons and Decreased Projections onto Basal Ganglia

**Figure 3:**
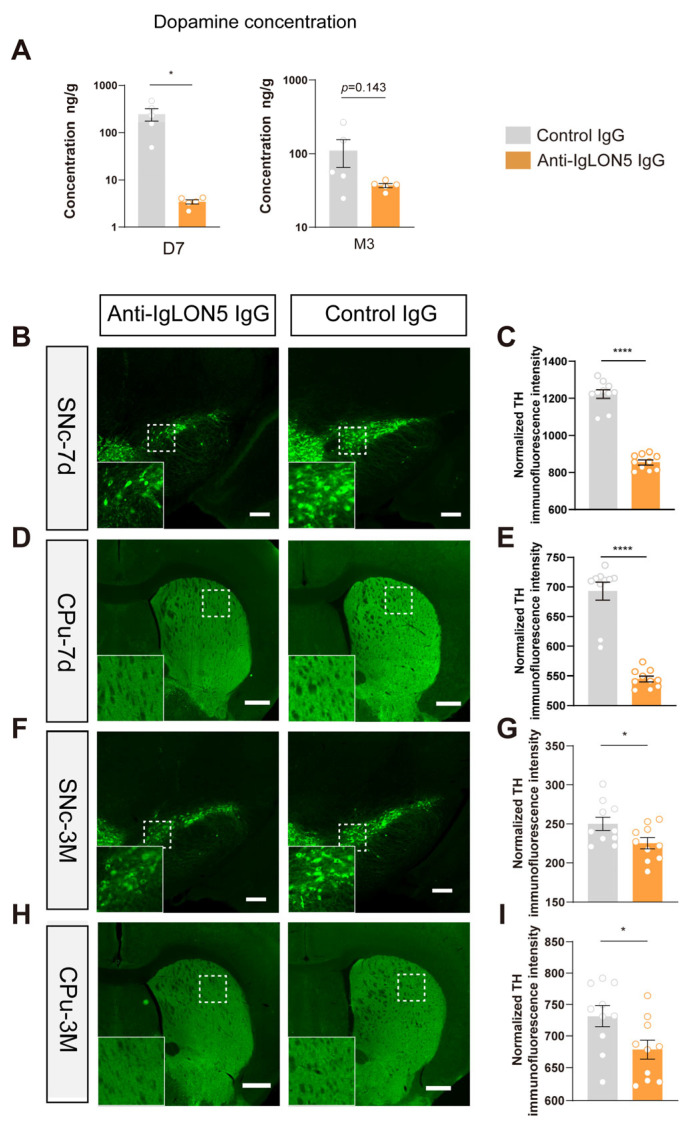
Anti-IgLON5 antibodies result in damage to the substantia nigra–striatum pathway. (**A**) Mice injected with anti-IgLON5 IgG showed a decrease in dopamine content in the striatum compared to the control group at 7 days (D7) (*n* = 5, *p* < 0.05, *t*-test). Both groups exhibited a decreasing trend in striatal dopamine content at 3 months (M3) (*n* = 5, *p* = 0.143, *t*-test). (**B**) Representative pictures of TH expression in the SNc of mice injected with patient IgG or control IgG killed on day 7. (**C**) Fluorescence intensity of TH staining in the SNc of mice injected with anti-IgLON5 IgG significantly decreased compared to the control group at 7 days (*n* = 10, *p* < 0.0001, *t*-test). (**D**) Representative pictures of TH expression in the caudate putamen (CPu) of mice injected with patient IgG or control IgG killed on day 7. (**E**) TH staining fluorescence intensity in the CPu region showed a significant decrease at 7 days in the anti-IgLON5 IgG group compared to the control group (*n* = 10, *p* < 0.0001, *t*-test). (**F**) Representative pictures of TH expression in the SNc of mice injected with patient IgG or control IgG killed on 3 months. (**G**) At 3 months, TH staining fluorescence intensity in the SNc region significantly decreased in the anti-IgLON5 IgG group compared to the control group (*n* = 10, *p* < 0.05, *t*-test). (**H**) Representative pictures of TH expression in the CPu of mice injected with patient IgG or control IgG killed on 3 months. (**I**) TH staining fluorescence intensity in the CPu region showed a significant decrease at 3 months in the anti-IgLON5 IgG group compared to the control group (*n* = 10, *p* < 0.05, *t*-test). * represents a *p*-value less than 0.05, and **** represents a *p*-value less than 0.0001. The white box represents a magnified view of the local area.

To investigate the underlying causes of motor impairment in mice following the injection of patient antibodies into the SNc, we used high-performance liquid chromatography (HPLC) to measure dopamine levels in the downstream structure of the classical nigrostriatal pathway, the dorsolateral striatum. We found that compared to the control group, mice injected with anti-IgLON5 antibodies exhibited a decrease in dopamine levels 7 days after injection (*n* = 5, *p* < 0.05). At the 3-month time point, the dopamine levels in the striatum of the anti-IgLON5 antibody-injected mice still showed a decreasing trend compared to the control group (*n* = 5, *p* = 0.143). This indicates that anti-IgLON5 antibodies have a direct or indirect impact on the dopaminergic pathway (Figure 3A).

To further validate the above hypothesis, we performed TH staining on the SNc and striatum of mice at 7 days and 3 months after antibody injection. The staining results revealed that, compared to the control group, the fluorescence intensity of dopaminergic neurons in the SNc was decreased in mice injected with anti-IgLON5 antibodies after 7 days (*n* = 10, *p* < 0.0001), while the fluorescence intensity of the projection fibers in the striatum was also reduced (*n* = 10, *p* < 0.0001). After 3 months of antibody injection, compared to the control group, the fluorescence intensity of dopaminergic neurons in the SNc continued to decrease in mice injected with anti-IgLON5 antibodies (*n* = 10, *p* < 0.05), and the fluorescence intensity of the projection fibers in the striatum was also reduced (*n* = 10, *p* < 0.05). These results suggest that the anti-IgLON5 antibodies can cause the death of TH neurons in the SNc (Figure 3B–I).

**Figure 4:**
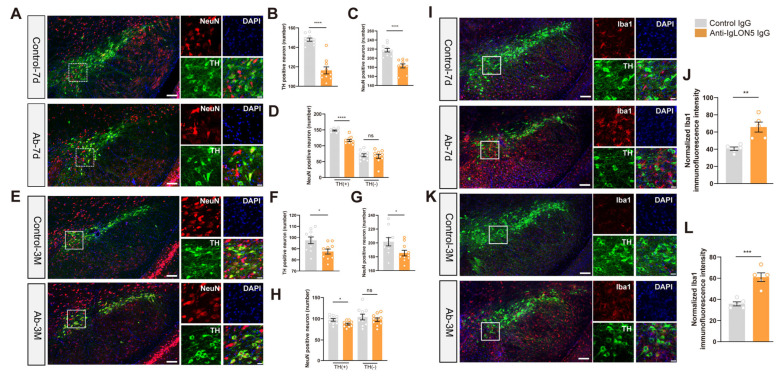
Death of TH neurons and activation of microglia. (**A**) Representative pictures of TH-positive and NeuN-positive neurons in the SNc of mice injected with patient IgG or control IgG killed on day 7. (**B**) The number of TH-positive cells in the SNc region of mice injected with anti-IgLON5 IgG decreased compared to the control group at 7 days post-injection (*n* = 10, *p* < 0.0001, *t*-test). (**C**) The number of NeuN-positive cells in the SNc region of mice injected with anti-IgLON5 IgG decreased compared to the control group at 7 days post-injection (*n* = 10, *p* < 0.0001, *t*-test). (**D**) At 7 days post-injection, there was a significant reduction in TH-positive neurons in the group injected with anti-IgLON5 antibodies compared to the control group (*n* = 10, *p* < 0.001), while TH-negative neurons remained unchanged. (**E**) Representative pictures of TH-positive and NeuN-positive in the SNc of mice injected with patient IgG or control IgG killed on 3 months. (**F**) The number of TH-positive cells in the SNc region of mice injected with anti-IgLON5 IgG decreased compared to the control group at 3 months post-injection (*n* = 10, *p* < 0.05, *t*-test). (**G**) The number of NeuN-positive cells in the SNc region of mice injected with anti-IgLON5 IgG decreased compared to the control group at 3 months post-injection (*n* = 10, *p* < 0.05, *t*-test). (**H**) At 3 months post-injection, there was a significant reduction in TH-positive neurons in the group injected with anti-IgLON5 antibodies compared to the control group (*n* = 10, *p* < 0.05), while TH-negative neurons remained unchanged. (**I**) Representative pictures of TH and Iba1 staining in the SNc of mice injected with patient IgG or control IgG killed on day 7. (**J**) The fluorescence intensity of Iba1 staining in the SNc of mice injected with anti-IgLON5 IgG increased significantly compared to the control group at 7 days (*n* = 5, *p* < 0.01, *t*-test). (**K**) Representative pictures of TH and Iba1 staining in the SNc of mice injected with patient IgG or control IgG killed on 3 months. (**L**) The fluorescence intensity of Iba1 staining in the SNc of mice injected with anti-IgLON5 IgG increased significantly compared to the control group at 3 months (*n* = 5, *p* < 0.001, *t*-test). * represents a *p*-value less than 0.05, ** represents a *p*-value less than 0.01, *** represents a *p*-value less than 0.001, and **** represents a *p*-value less than 0.0001. “ns” stands for no statistically significance. The white box represents a magnified view of the local area.

To confirm whether anti-IgLON5 antibodies cause the death of dopaminergic neurons in the SNc, we performed NeuN and TH staining in the SNc at both 7 days and 3 months after antibody injection. The results revealed that, after 7 days of anti-IgLON5 antibody injection, the number of NeuN-positive neurons in the SNc was reduced compared to the control group (*n* = 10, *p* < 0.0001), and there was also a noticeable decrease in the number of TH-positive neurons (*n* = 10, *p* < 0.0001). This indicates that the injection of anti-IgLON5 antibodies indeed leads to the death of dopaminergic neurons in the short term. Additionally, we compared the differences in TH-positive and TH-negative neurons between the group injected with anti-IgLON5 antibodies and the group injected with control IgG. The results demonstrated that there was a significant reduction in TH-positive neurons in the group injected with anti-IgLON5 antibodies compared to the control group (*n* = 10, *p* < 0.001), while TH-negative neurons remained unchanged. Furthermore, at 3 months after anti-IgLON5 antibodies injection, the number of NeuN-positive neurons in the SNc continued to decline compared to the control group (*n* = 10, *p* < 0.05), and there was a significant reduction in the number of TH-positive neurons compared to the control group (*n* = 10, *p* < 0.05). Additionally, there was a significant reduction in TH-positive neurons in the group injected with anti-IgLON5 antibodies compared to the control group (*n* = 10, *p* < 0.05), while TH-negative neurons remained unchanged. These results suggest that anti-IgLON5 antibodies specifically cause the death of TH neurons in SNc (Figure 4A–H).

### 3.3. The Long-Term Effects of Anti-IgLON5 Antibodies from Patients Leads to an Increase in p-Tau Protein

**Figure 5:**
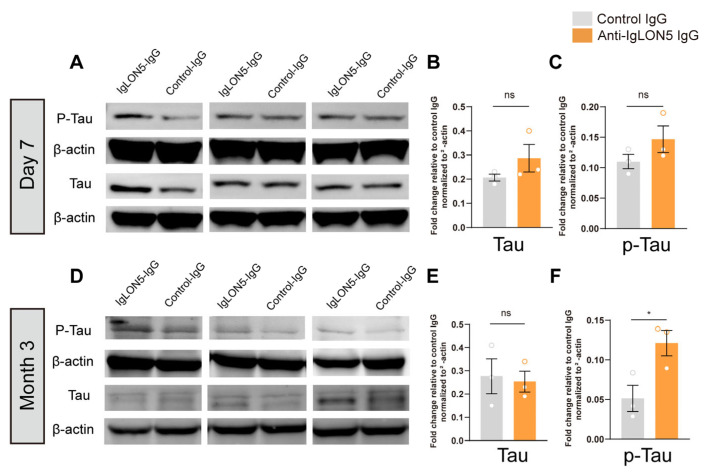
Expression levels of P-Tau and Tau proteins in the SNc. (**A**) Immunoblot analysis of P-Tau and Tau expression in the SNc of mice injected with patient IgG or control IgG killed on Day 7. (**B**,**C**) Seven days after antibody injection, there was no significant difference in the levels of Tau protein and p-Tau protein between the experimental group and the control group. (**D**) Immunoblot analysis of p-Tau and Tau expression in the SNc of mice injected with patient IgG or control IgG killed on 3 months. (**E**,**F**) After 3 months of antibody injection, there was no statistical difference in the levels of Tau protein between the anti-IgLON5 group and the control group, while the anti-IgLON5 group showed an increase in p-Tau levels, with *n* = 3, *p* < 0.05, *t*-test. * represents a *p*-value less than 0.05, and “ns” stands for no statistically significance.

Many clinical studies have shown that autopsies of patients with anti-IgLON5 antibody-associated neurological disorders reveal the deposition of phosphorylated Tau protein in certain regions of the midbrain, brainstem, and cerebellum. To investigate the effects of anti-IgLON5 antibodies on Tau protein, we selected two time points, 7 days and 3 months, and used WB to detect Tau and p-Tau in the SNc of the midbrain. The results showed no significant changes in Tau protein and p-Tau in the SNc after 7 days of antibody injection (*n* = 3, *p* > 0.05). However, after 3 months of injection, there was a significant increase in p-Tau in the SNc of mice in the anti-IgLON5 antibody group (*n* = 3, *p* < 0.05). This indicates that the long-term effects of IgLON5 antibodies lead to the deposition of p-Tau, while there are no significant differences in the short term (Figure 5).

### 3.4. The Presence of Anti-IgLON5 Antibodies from Patients Leads to Sustained Activation of Microglial Cells

To investigate the possible reasons for the death of dopaminergic neurons in the SNc following the injection of anti-IgLON5 antibodies, we performed immunofluorescence staining of Iba1 in the SNc. Based on our hypothesis, the death of dopaminergic neurons is associated with the function of microglial cells. The results showed an increased Iba1 staining in the SNc of mice injected with anti-IgLON5 antibodies at both 7 days (*n* = 5, *p* < 0.01) and 3 months (*n* = 5, *p* < 0.001). This indicates that the injection of anti-IgLON5 antibodies into the SNc leads to sustained activation of microglial cells in the SNc (Figure 4I–L).

### 3.5. Whole-Genome Transcriptomic Analyses in SNc

We conducted RNA-seq analysis on mice injected with patient IgG and control IgG to explore the potential molecular mechanisms at day 7 and month 3 after continuous IgG injections. At day 7, we identified 90 differentially expressed genes (DEGs) between mice injected with anti-IgLON5 IgG and control IgG, with 72 genes upregulated and 18 genes downregulated (*p*-value < 0.05, absolute log2 fold change > 1). To visualize the overall dispersion of genes and filter the DEGs, we constructed a volcano plot. The distribution of DEGs was reflected through the Gene Ontology (GO) enrichment scatter plot, including three aspects: biological processes, cellular components, and molecular functions. We then focused on the DEGs with relatively higher expression levels and different functions. As expected, most of the differentially expressed genes were associated with inflammation and immune responses, such as Cd69, Cd274, IL2Rg, and Cxcl13. Some DEGs also revealed roles in cell adhesion and the cytoskeleton. Additionally, there were DEGs related to neurotransmitter transporters, such as Slc6a2, Slc18a3, and Slc6a5, which may be involved in changes in neurotransmitters.

**Figure 6:**
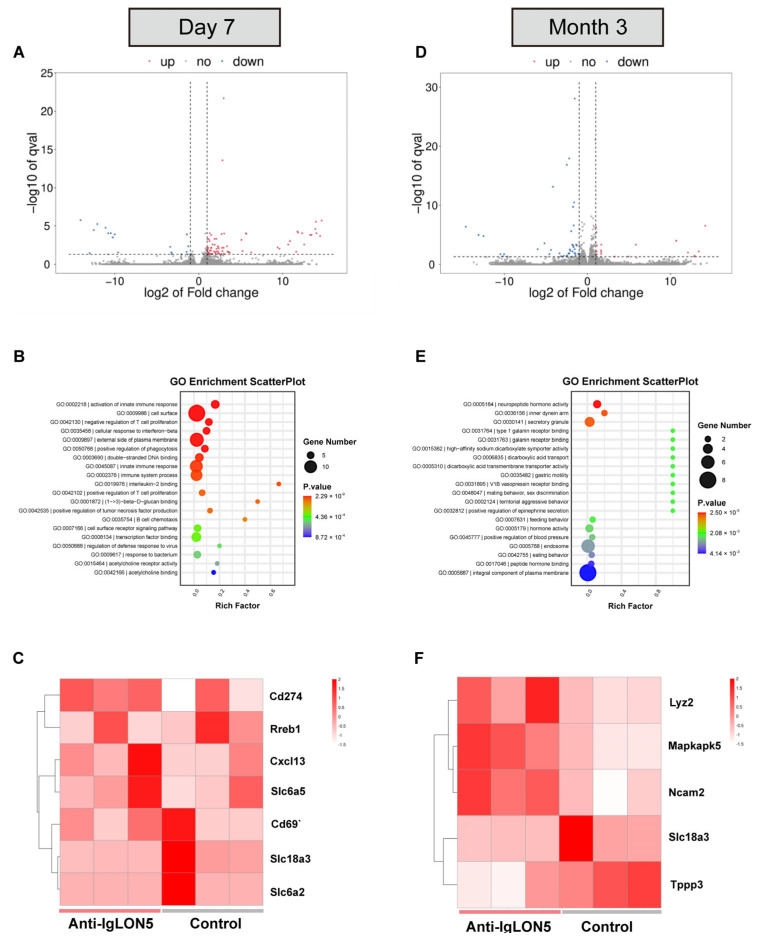
Whole-genome transcriptomic analyses. (**A**) Volcano plot of differentially expressed genes between anti-IgLON5-IgG-injected mice and HC-IgG-injected mice at day 7. Downregulation and upregulation are shown in blue and red dots, respectively. (**B**) Statistics of GO enrichment on day 7. Rich factor = S gene number/B gene number. The size of dots represents the S gene number, and the color of dots represents the *p*-value. S gene, significantly differentially expressed gene annotated as specific GO term; B gene, gene annotated as a specific GO term. (**C**) Cluster analysis of high expression levels of differentially expressed genes on day 7. There are three main categories: biological processes, cellular components, and molecular functions. (**D**) Volcano plot of differentially expressed genes between anti-IgLON5-IgG-injected mice and HC-IgG-injected mice at 3 months. (**E**) Statistics of GO enrichment in 3 months. (**F**) Cluster analysis of high expression levels of differentially expressed genes in 3 months. There are three main categories: neurotransmitter activity, secretory granules, and microtubule proteins.

However, at the third month, we found 62 DEGs between mice injected with anti-IgLON5 IgG and control IgG, with 19 genes upregulated and 43 genes downregulated. GO enrichment analysis showed that most of the differentially expressed genes were associated with neurotransmitter activity, secretory granules, microtubule proteins, and other factors, such as Gabrb2, Slc18a3, Tppp3, Lyz2, and Ncam2. The RNA-seq results indicated that the short-term effects of the antibodies were related to inflammation and immune responses, while the long-term effects primarily affected neurotransmitter activity, secretory granules, and microtubule proteins. Further exploration of the underlying mechanisms can focus on the differential roles of anti-IgLON5 antibodies in the short and long term (Figure 6).

## 4. Discussion

In this study, we first established a motor impairment model of anti-IgLON5 disease by directly injecting anti-IgLON5 antibodies into the mouse SNc. We found that anti-IgLON5 antibodies have long-term effects, leading to sustained motor impairment. This may be related to the impact of anti-IgLON5 antibodies on the nigrostriatal pathway. Additionally, for the first time, we discovered in vivo that anti-IgLON5 antibodies can induce the deposition of phosphorylated Tau protein in an animal model.

### 4.1. The Movement Impairment Mediated by Anti-IgLON5 Antibodies Persists in the Long Term

Similar to neurodegenerative diseases, anti-IgLON5 disease often exhibits a chronic course, which is different from anti-NMDA receptor encephalitis and typically presents rapidly within days or weeks [27]. Considering that in animal models of anti-NMDAR encephalitis, behavioral impairments can recover without treatment after a 14-day cessation of antibody injection [28,29], whereas, in the animal model of anti-IgLON5 disease, motor impairment persists even after a 3-month cessation of antibody injection, we believe this indicates a long-term chronic effect of the antibodies.

We observed that injecting anti-IgLON5 antibodies causes the death of dopaminergic neurons in the SNc. The phenomenon of neuronal death induced by anti-IgLON5 antibodies was consistent with findings from other studies. Notably, significant cell death was observed when induced pluripotent stem cell cultures were co-cultured with anti-IgLON5 IgG for 21 and 35 days [10]. Similar neuronal death was observed in vivo when a continuous infusion of anti-IgLON5 antibodies was administered to the hippocampal CA1 region, leading to the death of neurons [4]. This neuronal damage may be associated with disruptions in the cellular cytoskeleton and certain neuroinflammatory responses [4].

### 4.2. Motor Impairment Is Associated with the Nigrostriatal Dopaminergic Pathway

SPECT imaging in patients with anti-IgLON5 disease reveals a decrease in striatal uptake [6,7,8]. Combining the MRI findings in the previously reported alterations in striatal metabolism, it is speculated that the motor symptom phenotype of anti-IgLON5 disease may be related to the dopaminergic pathway in the nigrostriatal system.

After injecting anti-IgLON5 antibodies into the SNc, a reduction in tyrosine hydroxylase (TH) neurons and their corresponding projections to the striatum was observed after 7 days of cessation of antibody infusion. Simultaneously, a decrease in striatal dopamine was observed. The nigrostriatal pathway is a crucial pathway in the brain that regulates movement. Damage to the dopaminergic neurons in the SNc or alterations in striatal dopamine levels can lead to motor impairment.

### 4.3. Long-Term Effects of IgLON5 Antibodies Lead to Neurodegenerative Changes

Among autoimmune encephalitis disorders, anti-IgLON5 disease is a rare autoimmune encephalitis characterized by neurodegenerative pathology. Gelpi et al. summarized the typical neuropathological features of anti-IgLON5-related Tau proteinopathy [5]. Autopsy results of patients with anti-IgLON5 disease showed high levels of phosphorylated Tau protein, including 3R (three-repeat) and 4R (four-repeat) isoforms, primarily affecting the hypothalamus and brainstem. The underlying mechanisms of how anti-IgLON5 antibodies induce Tau protein deposition and when Tau protein deposition begins have not been elucidated in basic research.

Our study provides some clues to these questions. We observed the deposition of phosphorylated Tau protein at 3 months after discontinuation of anti-IgLON5 antibody infusion, while this phenomenon was not observed at 7 days after discontinuation. Therefore, the deposition of phosphorylated Tau protein appears as a long-term outcome of antibody action and may not necessarily require continuous stimulation and the presence of anti-IgLON5 antibodies. These findings suggest that different treatment approaches may be needed in the early and late stages of anti-IgLON5 disease to improve the long-term prognosis for patients.

This study primarily observed the characteristics and duration of anti-IgLON5 antibody-mediated movement disorder, partially explaining their relationship with neurodegeneration. It was discovered that the movement disorder may be associated with impaired dopaminergic pathways. However, there is still a lack of in-depth exploration into the mechanisms underlying anti-IgLON5 antibody-mediated movement disorders. Additionally, this study only utilized purified anti-IgLON5 antibodies from a single patient’s serum sample, and future studies can incorporate a mixed formulation from multiple patients. Furthermore, this study did not investigate the mechanisms of sleep disorders in anti-IgLON5 disease. The next step will involve conducting research in this area.

## 5. Limitation

Due to the rarity of anti-IgLON5 disease, we were able to obtain serum samples from only one patient, which were subsequently purified and used in the mouse experiments. In the future, we aim to collect serum antibodies from more patients. Additionally, the purification of total IgG from a serum has inherent limitations. We are in the process of developing a method to selectively purify anti-IgLON5 antibodies from total IgG. Furthermore, the passive transfer animal model has certain limitations. To address this, we plan to enhance the animal model of this disease by adopting an active immunization method in further research.

## 6. Conclusions

This study reports several novel findings regarding the anti-IgLON5 antibody-mediated movement impairment phenotype through SNc. (1) It has been firstly observed that anti-IgLON5 antibodies can cause relatively long-lasting effects, with motor impairment persisting for 3 months in passive transfer animal models. (2) The motor impairment associated with anti-IgLON5 antibodies may be related to the nigrostriatal dopaminergic pathway. (3) The deposition of p-Tau has been observed in vivo for the first time, indicating long-term exposure to anti-IgLON5 antibodies can lead to neurodegenerative changes. These findings support the pathogenic role of anti-IgLON5 antibodies, which exhibit both neuroimmune and neurodegenerative characteristics. They provide a more detailed understanding of how these antibodies contribute to the clinical manifestations observed in patients.

## Data Availability

The data that support the findings of this study are available from the corresponding author upon reasonable request.

## Supplementary Materials

The following supporting information can be downloaded at: https://www.mdpi.com/article/10.3390/biomedicines11092483/s1, Figure S1: Titer, Subtypes, and CO-IP detection of anti-IgLON5 antibodies in the patient serum. (A) Antibodies against IgLON5 were detected in the serum using an immunofluorescence assay. The titer of anti-IgLON5 antibodies is 1:1000 in the serum sample. Images show the patient sample and the positive and negative control. (B) As the gold standard, the Co-IP method validated the presence of anti-IgLON5 antibodies in the serum. (C) Subclass of anti-IgLON5 IgG in serum samples of our patient. The titers for various subtypes were as follows: IgG1 1:100, IgG4 1:100, IgG2 1:30.

## References

[1] Sabater L., Gaig C., Gelpi E., Bataller L., Lewerenz J., Torres-Vega E., Contreras A., Giometto B., Compta Y., Embid C. (2014). A novel non-rapid-eye movement and rapid-eye-movement parasomnia with sleep breathing disorder associated with antibodies to IgLON5: A case series, characterisation of the antigen, and post-mortem study. Lancet Neurol..

[2] Zhang Y.H., Ni Y., Gao Y.N., Shen D.D., He L., Yin D., Meng H.Y., Zhou Q.M., Hu J., Chen S. (2023). Anti-IgLON5 disease: A novel topic beyond neuroimmunology. Neural Regen. Res..

[3] Ni Y., Shen D., Zhang Y., Song Y., Gao Y., Zhou Q., He L., Yin D., Wang Y., Song F. (2022). Expanding the clinical spectrum of anti-IgLON5 disease: A multicenter retrospective study. Eur. J. Neurol..

[4] Ni Y., Feng Y., Shen D., Chen M., Zhu X., Zhou Q., Gao Y., Liu J., Zhang Q., Shen Y. (2022). Anti-IgLON5 antibodies cause progressive behavioral and neuropathological changes in mice. J. Neuroinflamm..

[5] Gelpi E., Hoftberger R., Graus F., Ling H., Holton J.L., Dawson T., Popovic M., Pretnar-Oblak J., Hogl B., Schmutzhard E. (2016). Neuropathological criteria of anti-IgLON5-related tauopathy. Acta Neuropathol..

[6] Montojo T., Piren V., Benkhadra F., Codreanu A., Diederich N.J. (2017). Gaze Palsy, Sleep and Gait Disorder, as Well as Tako-Tsubo Syndrome in a Patient with IgLON5 Antibodies. Mov. Disord. Clin. Pract..

[7] Fuseya K., Kimura A., Yoshikura N., Yamada M., Hayashi Y., Shimohata T. (2020). Corticobasal Syndrome in a Patient with Anti-IgLON5 Antibodies. Mov. Disord. Clin. Pract..

[8] Gonzalez-Avila C., Casado L., Muro Garcia I., Villacieros-Alvarez J., Vivancos J., Quintas S. (2021). Altered ioflupane single-photon emission computed tomography in anti-IgLON5 disease: A new case mimicking probable progressive supranuclear palsy and review of the literature. Eur. J. Neurol..

[9] Haitao R., Yingmai Y., Yan H., Fei H., Xia L., Honglin H., Chaiyan L., Stocker W., Liying C., Hongzhi G. (2016). Chorea and parkinsonism associated with autoantibodies to IgLON5 and responsive to immunotherapy. J. Neuroimmunol..

[10] Ryding M., Gamre M., Nissen M.S., Nilsson A.C., Okarmus J., Poulsen A.A.E., Meyer M., Blaabjerg M. (2021). Neurodegeneration Induced by Anti-IgLON5 Antibodies Studied in Induced Pluripotent Stem Cell-Derived Human Neurons. Cells.

[11] Haitao R., Huiqin L., Tao Q., Xunzhe Y., Xiaoqiu S., Wei L., Jiewen Z., Liying C., Hongzhi G. (2017). Autoimmune encephalitis associated with vitiligo?. J. Neuroimmunol..

[12] Zhang W., Niu N., Cui R. (2016). Serial 18F-FDG PET/CT Findings in a Patient with IgLON5 Encephalopathy. Clin. Nucl. Med..

[13] DeLong M.R. (1990). Primate models of movement disorders of basal ganglia origin. Trends Neurosci..

[14] Forde N.J., Zwiers M.P., Naaijen J., Akkermans S.E.A., Openneer T.J.C., Visscher F., Dietrich A., Buitelaar J.K., Hoekstra P.J. (2017). Basal ganglia structure in Tourette’s disorder and/or attention-deficit/hyperactivity disorder. Mov. Disord..

[15] Gerfen C.R., Surmeier D.J. (2011). Modulation of striatal projection systems by dopamine. Annu. Rev. Neurosci..

[16] Surmeier D.J., Ding J., Day M., Wang Z., Shen W. (2007). D1 and D2 dopamine-receptor modulation of striatal glutamatergic signaling in striatal medium spiny neurons. Trends Neurosci..

[17] Nambu A. (2004). A new dynamic model of the cortico-basal ganglia loop. Prog. Brain Res..

[18] Alexander G.E., DeLong M.R., Strick P.L. (1986). Parallel organization of functionally segregated circuits linking basal ganglia and cortex. Annu. Rev. Neurosci..

[19] Giannoccaro M.P., Menassa D.A., Jacobson L., Coutinho E., Prota G., Lang B., Leite M.I., Cerundolo V., Liguori R., Vincent A. (2019). Behaviour and neuropathology in mice injected with human contactin-associated protein 2 antibodies. Brain.

[20] Li J., Lu C., Gao Z., Feng Y., Luo H., Lu T., Sun X., Hu J., Luo Y. (2020). SNRIs achieve faster antidepressant effects than SSRIs by elevating the concentrations of dopamine in the forebrain. Neuropharmacology.

[21] Zeng Y., Luo H., Gao Z., Zhu X., Shen Y., Li Y., Hu J., Yang J. (2021). Reduction of prefrontal purinergic signaling is necessary for the analgesic effect of morphine. iScience.

[22] Rojas-Carvajal M., Brenes J.C. (2020). Acute stress differentially affects grooming subtypes and ultrasonic vocalisations in the open-field and home-cage test in rats. Behav. Process..

[23] Paraskevas N., Ayari R., Malikov S., Mollo M., Branchereau P., Hut F., Branchereau A. (2006). ‘Pole test’ measurements in critical leg ischaemia. Eur. J. Vasc. Endovasc. Surg..

[24] Orenduff M.C., Rezeli E.T., Hursting S.D., Pieper C.F. (2021). Psychometrics of the Balance Beam Functional Test in C57BL/6 Mice. Comp. Med..

[25] Liu Y., Zhang Y., Zheng X., Fang T., Yang X., Luo X., Guo A., Newell K.A., Huang X.F., Yu Y. (2018). Galantamine improves cognition, hippocampal inflammation, and synaptic plasticity impairments induced by lipopolysaccharide in mice. J. Neuroinflamm..

[26] Taleb A., Zhou Y.P., Meng L.T., Zhu M.Y., Zhang Q., Naveed M., Li L.D., Wang P., Zhou Q.G., Meng F. (2021). New application of an old drug proparacaine in treating epilepsy via liposomal hydrogel formulation. Pharmacol. Res..

[27] Werner J., Jelcic I., Schwarz E.I., Probst-Muller E., Nilsson J., Schwizer B., Bloch K.E., Lutterotti A., Jung H.H., Schreiner B. (2021). Anti-IgLON5 Disease: A New Bulbar-Onset Motor Neuron Mimic Syndrome. Neurol. Neuroimmunol. Neuroinflamm..

[28] Garcia-Serra A., Radosevic M., Pupak A., Brito V., Rios J., Aguilar E., Maudes E., Arino H., Spatola M., Mannara F. (2020). Placental transfer of NMDAR antibodies causes reversible alterations in mice. Neurol. Neuroimmunol. Neuroinflamm..

[29] Carceles-Cordon M., Mannara F., Aguilar E., Castellanos A., Planaguma J., Dalmau J. (2020). NMDAR Antibodies Alter Dopamine Receptors and Cause Psychotic Behavior in Mice. Ann. Neurol..

