# Supplementary material for: Purified Serum IgG from a Patient with Anti-IgLON5 Antibody Cause Long-Term Movement Disorders with Impaired Dopaminergic Pathways in Mice
Source: Biomedicines. 2023 Sep 7;11(9):2483. doi: 10.3390/biomedicines11092483 (PMC10526147; doi:10.3390/biomedicines11092483)
Supplement: Supplementary file 1 [file biomedicines-11-02483-s001.zip › biomedicines-2535581-supplementary.pdf]

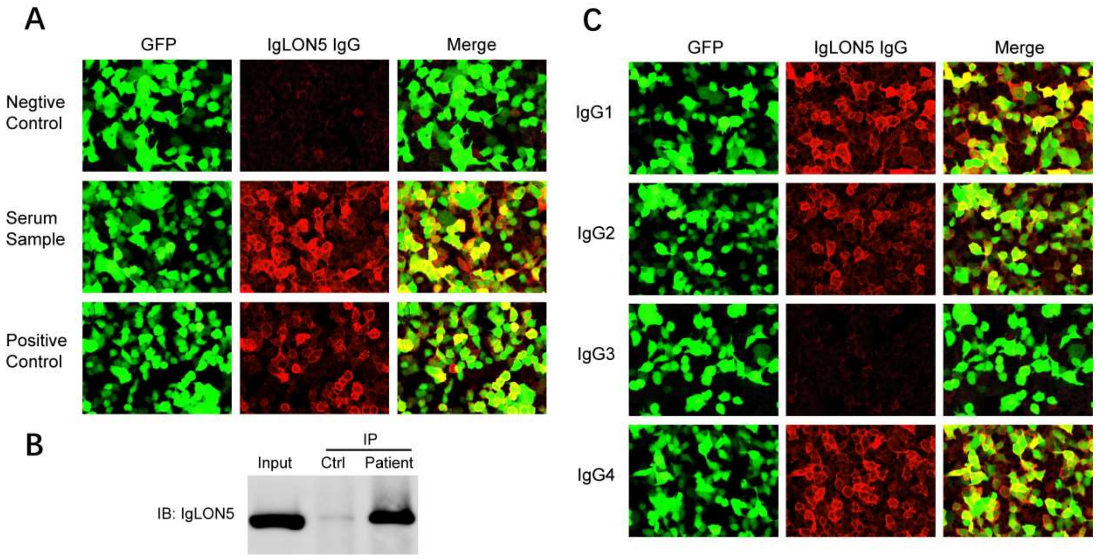

Supplementary figure S1 Titer, Subtypes, and CO-IP detection of anti-IgLON5 antibodies in the patient serum. (A) Antibodies against IgLON5 were detected in the serum using an immunofluorescence assay. The titer of anti-IgLON5 antibodies is 1:1000 in the serum sample. Images show the patient sample and the positive and negative control. (B) As the gold standard, the Co-IP method validated the presence of anti-IgLON5 antibodies in the serum. (C) Subclass of anti-IgLON5 IgG in serum samples of our patient. The titers for various subtypes were as follows: IgG1 1:100, IgG4 1:100, IgG2 1:30.
